# Alcohol consumption and the risk of morbidity and mortality for different stroke types - a systematic review and meta-analysis

**DOI:** 10.1186/1471-2458-10-258

**Published:** 2010-05-18

**Authors:** Jayadeep Patra, Benjamin Taylor, Hyacinth Irving, Michael Roerecke, Dolly Baliunas, Satya Mohapatra, Jürgen Rehm

**Affiliations:** 1 Centre for Addiction and Mental Health, Toronto, Ontario Canada; 2 Dalla Lana School of Public Health, University of Toronto, Toronto, Canada; 3 Technische Universität Dresden, Dresden, Germany; 4 Dept. of Psychiatry, University of Toronto, Toronto, Canada

## Abstract

**Background:**

Observational studies have suggested a complex relationship between alcohol consumption and stroke, dependent on sex, type of stroke and outcome (morbidity vs. mortality). We undertook a systematic review and a meta-analysis of studies assessing the association between levels of average alcohol consumption and relative risks of ischemic and hemorrhagic strokes separately by sex and outcome. This meta-analysis is the first to explicitly separate morbidity and mortality of alcohol-attributable stroke and thus has implications for public health and prevention.

**Methods:**

Using Medical Subject Headings (alcohol drinking, ethanol, cerebrovascular accident, cerebrovascular disorders, and intracranial embolism and thrombosis and the key word stroke), a literature search of MEDLINE, EMBASE, CINAHL, CABS, WHOlist, SIGLE, ETOH, and Web of Science databases between 1980 to June 2009 was performed followed by manual searches of bibliographies of key retrieved articles. From twenty-six observational studies (cohort or case-control) with ischemic or hemorrhagic strokes the relative risk or odds ratios or hazard ratios of stroke associated with alcohol consumption were reported; alcohol consumption was quantified; and life time abstention (manually estimated where data for current abstainers were given) was used as the reference group. Two reviewers independently extracted the information on study design, participant characteristics, level of alcohol consumption, stroke outcome, control for potential confounding factors, risk estimates and key criteria of study quality using a standardized protocol.

**Results:**

The dose-response relationship for hemorrhagic stroke had monotonically increasing risk for increasing consumption, whereas ischemic stroke showed a curvilinear relationship, with a protective effect of alcohol for low to moderate consumption, and increased risk for higher exposure. For more than 3 drinks on average/day, in general women had higher risks than men, and the risks for mortality were higher compared to the risks for morbidity.

**Conclusions:**

These results indicate that heavy alcohol consumption increases the relative risk of any stroke while light or moderate alcohol consumption may be protective against ischemic stroke. Preventive measures that should be initiated are discussed.

## Background

Stroke is an international health problem with high associated human and economic costs. Among adults, it is the second-leading cause of death worldwide, and ranks fourth in overall disease burden. Recent trend analysis showed that stroke incidence and associated deaths appears to be rising, particularly in economically emerging countries [1,2]. Recent projections suggest that, without intervention, the number of deaths from stroke will continue rising, to 6.5 million in 2015 and 7.8 million by 2030, with the vast bulk/majority in poor countries [3]. Risk factor identification and analysis is therefore a high priority in various fields of stroke research and will be important in containing and reducing this heavy disease burden.

It is well accepted that heavy alcohol consumption has been linked to an increased risk of ischemic stroke [4] and hemorrhagic stroke [4-6]. However, some studies of moderate alcohol and stroke reported a protective effect of alcohol at these doses [4,7-9] while others have found that moderate consumption increases the overall risk [5,10]. What's more, the only meta-analyses on the subject only focus on mortality from stroke, even though morbidity is much more common for this event and alcohol may have a differential role in fatal and non-fatal events [11,12]. This study will attempt to systematically estimate the impact of alcohol for stroke separately by event outcome and provide estimates by level of alcohol consumption via meta-analysis.

## Methods

### Search Strategy

We conducted a systematic literature search of MEDLINE, EMBASE, CINAHL, CABS, WHOlist, SIGLE, ETOH, and Web of Science for relevant original papers from January 1980 to first week of June 2009. We used the following keywords and medical subject headings to identify relevant articles in electronic databases: (alcohol or ethanol) AND (stroke* or cerebrovascular* or intracranial embolism or thrombosis) AND (case or cohort or ratio or risk* or prospective* or follow*). No language restrictions were applied. Generally, studies were eligible for inclusion if they were original publications (we excluded letters, editorials, conference abstracts, reviews, and comments) of case-control and cohort studies reporting incidence, hazard ratio (HR), relative risk (RR) or odds ratio (OR) of alcohol consumption in comparison to no alcohol consumption. In addition, bibliographies of key retrieved articles, relevant reviews and meta-analyses were hand searched.

### Inclusion and exclusion criteria

To be included in our meta-analysis, a published study had to meet the following criteria: (1) had to be an original research study (not a review); (2) cohort or case-control study in which medically confirmed ischemic or hemorrhagic stroke were end points (i.e., not self-reported endpoint); (3) reporting of RRs or ORs or HRs (or data to calculate these risks) of stroke associated with alcohol consumption compared to abstention; (4) having three or more alcohol drinking exposure groups (i.e., dose-response information was required).

### Data Extraction

All data were independently abstracted by means of a standardized protocol. Study characteristics recorded were as follows: title, lead author name, year, and source of publication, country of origin, study design (cohort study or case-control study), characteristics of the study population (sample size; sampling methods; and distribution of age, average age at baseline, sex, and ethnicity), measures of outcome and exposure (the number of cases at each exposure level, the total population at risk at each exposure level), duration of follow-up (for prospective cohort studies), confounding factors controlled for by matching or adjustment, and the RR (or OR or HR) and the corresponding lower and upper 95% confidence intervals of stroke types, assessment of current or life time abstention, and level of alcohol consumption. RRs were abstracted by sex, subtype of stroke (ischemic or hemorrhagic), end point incidence (mortality, morbidity), and level of alcohol consumption. If studies only reported results for both sexes combined, the same results were applied to both female and male datasets. Similarly, if combined results were reported for mortality and morbidity studies, the same results were applied to both mortality and morbidity datasets.

To ensure accuracy in data abstraction, five included and five excluded studies were randomly chosen to be abstracted independently by a co-author (HI). There was a 93.6% overall agreement between the 2 independent raters. For data abstraction the raters agreed on 92.0% of possible data points, and 95.2% with respect to quality scoring (data not shown). Where disagreements existed, both authors discussed the discrepancy until a consensus was reached.

### Standardization of alcohol consumption

Where consumption was reported in drinks and not grams, the gram pure alcohol equivalent described in the article was used as a conversion factor if stated, and if not, conversion from standard drinks was based on geography: for Canada 13.6 grams, the UK 8 grams, the USA 12 grams and in both New Zealand and Australia 10 grams of pure alcohol were assumed based on the literature [13]. For all other countries without clear standard drink specifications 12 grams pure alcohol was used. For those studies [4,6,9,10,14-18] that did not report measures of association separately by sex, the estimates were used for men as well as women. Information on alcohol consumption was extracted. When ranges of alcohol consumption were given, the midpoint was taken. In cases where no upper bound for the highest category was given, 3/4 of the length of the immediate previous category range was added to the lower bound and was used as the measure.

### Correction for lifetime abstention

If a study only reported RR relative to current abstention, the risk estimate and 95% confidence intervals were adjusted to lifetime abstention. Studies reporting RR of ex-drinkers relative to lifetime abstainers [14,15,19-24] were grouped together by sex and a pooled RR for ex-drinkers was determined. In addition, the ratio of ex-drinkers relative to lifetime abstainers in this pooled estimate was calculated. For those studies only reporting current abstention, the proportion of lifetime abstainers and ex-drinkers was estimated based on the ratio of ex-drinkers previously calculated and the RRs in each article were adjusted based on the pooled RR. This was done to avoid the "sick-quitter effect", a situation where previously heavy drinkers who had stopped drinking because of health reasons confound the true RR for abstainers by artificially inflating the lifetime abstainers' RR for stroke, although people might have stopped drinking for other reasons as well [25].

### Statistical Analysis

The dose-response relationship between alcohol consumption and risk of strokes was assessed with random effects meta-regression models. Based on previously published research, the association between alcohol consumption and stroke was expected to be both linear and non-linear, depending on the subtype. Alcohol consumption was modeled as a continuous variable using the fractional polynomial method [26] to estimate the relationship between alcohol consumption and the logarithmized RR of stroke subtypes. In order to be flexible in fitting the best model, we conducted the meta-regression using linear as well as first-order and second-order fractional polynomials with powers of -2, -1, -0.5, 0, 0.5, 1, 2, and 3 to estimate a best fitting curve to the data. All models were fitted in STATA using the GLST function separately for sex and outcome. Best-fit curves or lines were assessed using standard goodness-of-fit statistics with an emphasis on reduced deviance (gain) compared with the quadratic model. Comparisons of curves to determine the best fit were made using a Chi-square distribution, as recommended by Royston [27].

Statistical heterogeneity among studies was assessed using both the Cochrane Q test and the I^2 ^statistic. For the Q-statistic, a p-value of < 0.10 was considered to be representative of statistically significant heterogeneity. I^2 ^ranges between 0% and 100% and describes the percentage of total variation across studies that is due to heterogeneity. A value of zero indicates no observed heterogeneity, and larger values show increasing heterogeneity [28,29]. Publication bias was assessed by visual inspection of Begg's funnel plot [30] and by applying two statistical tests: Begg-Mazumdar adjusted rank correlation test [31], and the Egger regression asymmetry test [30]. A p-value of < 0.10 was considered to be representative of statistically significant publication bias.

All analyses were conducted using STATA software version 10.1 [32].

## Results

**Figure 1:**
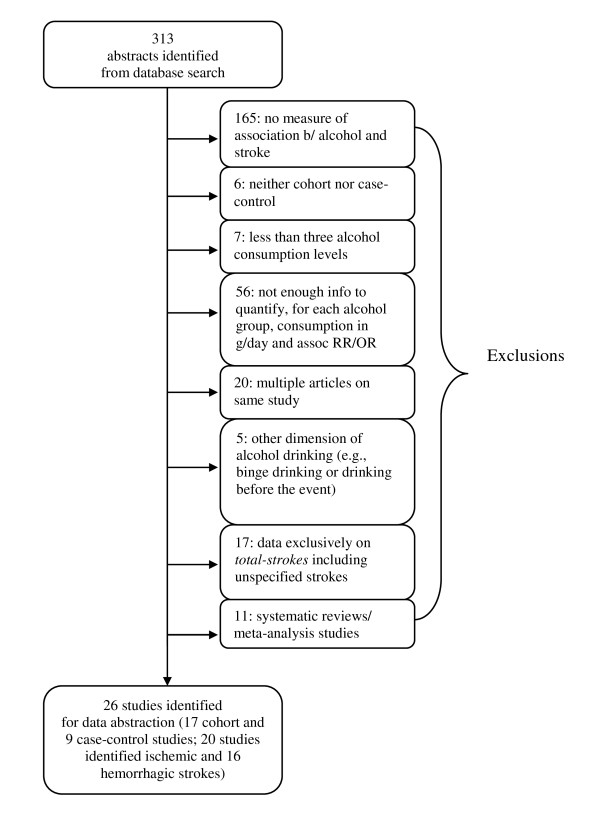
**Results of systematic review of the relationship between alcohol and stroke subtypes**.

We identified 26 studies that met the inclusion criteria outlined in Figure 1. There were 17 cohort studies [5,14-16,19-24,33-39] and 9 case-control studies [4,6,8-10,17,18,40,41] (See Figure 1). Four previous systematic reviews [12,42-44] and seven meta-analyses [45-51] were identified and excluded; some meta-analytic studies were published either as an iteration [46] or as an update [43] from past studies.

### Study Characteristics

The characteristics of the study subjects and design of the studies are presented in Additional files 1 and 2. The number of subjects in the cohort studies ranged from 1621 in the study by Kiyohara et al [16] to 107,137 in the study by Klatsky et al [19]. Among all cohort studies, 13 studies [14,16,19-21,23,24,34-38] reported ischemic stroke, and 12 studies [5,15,16,19,22-24,33,34,36,38,39]; reported hemorrhagic stroke as the outcome. Similarly, 15 studies [5,15,16,20-24,33-39] had mortality as the end point; whereas 11 studies [5,14,16,19-21,23,33,34,36,37] had morbidity as end point. The follow-up period ranged from 4 to 30 years.

The number of case subjects enrolled in case-control studies ranged from 82 in the study by Henrich & Horwitz [40] to 677 in the study by Sacco and colleagues [18] and the corresponding number of control subjects ranged from 153 in the study by Palomaki & Kaste [41] to 1139 in the study by Sacco and colleagues [18]. Seven studies [4,8-10,18,40,41] collected data on ischemic stroke and 4 studies [4,6,9,17] collected data on hemorrhagic stroke. Three studies [6,17,18] had mortality as the end point and 9 studies [4,6,8-10,17,18,40,41] had morbidity as an end point.

### Heterogeneity assessment by stroke type

#### Hemorrhagic stroke

**Figure 2:**
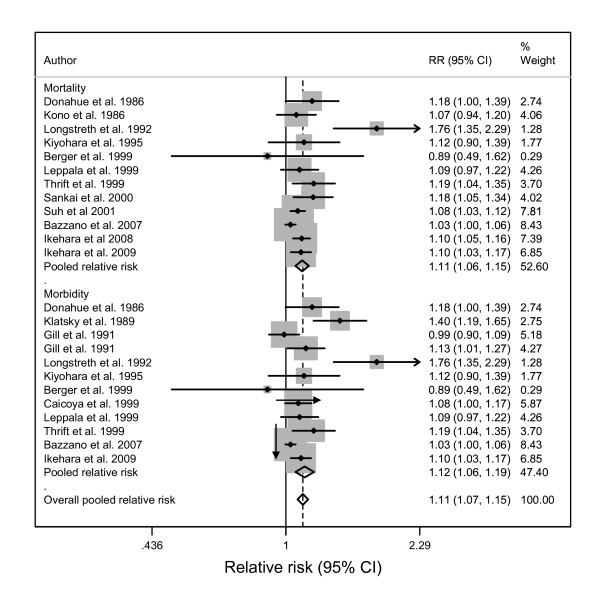
**Forest plot of risk estimates for alcohol consumption related to haemorrhagic stroke of men by endpoint (15 studies)**.

For both men and women morbidity datasets, there was significant heterogeneity between studies (men: Q-statistic (df) = 87.7 (39), p = 0.000; I^2 ^= 56, 95% CI: 37-69; women: Q-statistic = 34.0, p = 0.005; I^2 ^= 53, 95% CI: 18-73) (See Figures 2). Publication bias was not detected by either Begg's (p > 0.05) or Egger's (p > 0.05) tests for either men or women. Moreover, the "trim and fill" method did not produce any additional studies, further corroborating that there was no publication bias.

**Figure 3:**
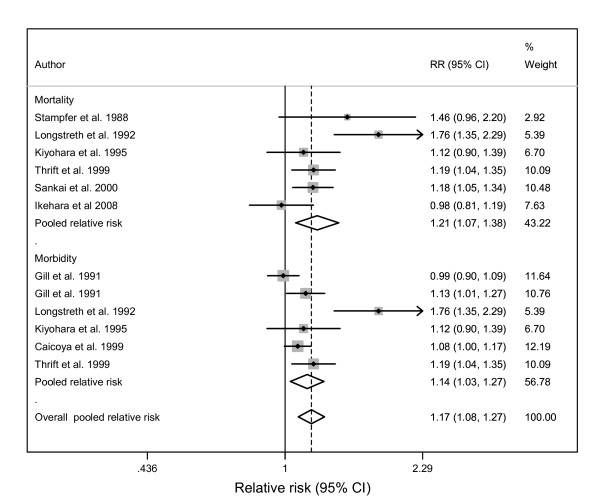
**Forest plot of risk estimates for alcohol consumption related to haemorrhagic stroke of women by endpoint (8 studies)**.

Similarly, in the mortality datasets, there was significant heterogeneity (Q-statistic = 63.5(38), p = 0.006; I^2 ^= 40, 95% CI: 12-59) among men but not among women (Q-statistic = 21.0 (16), p = 0.179; I^2 ^= 24, 95% CI: 0-58) (See Figure 3). No publication bias was detected by either Begg's (p > 0.05) or Egger's (p > 0.05) tests or the "trim and fill" method.

#### Ischemic stroke

**Figure 4:**
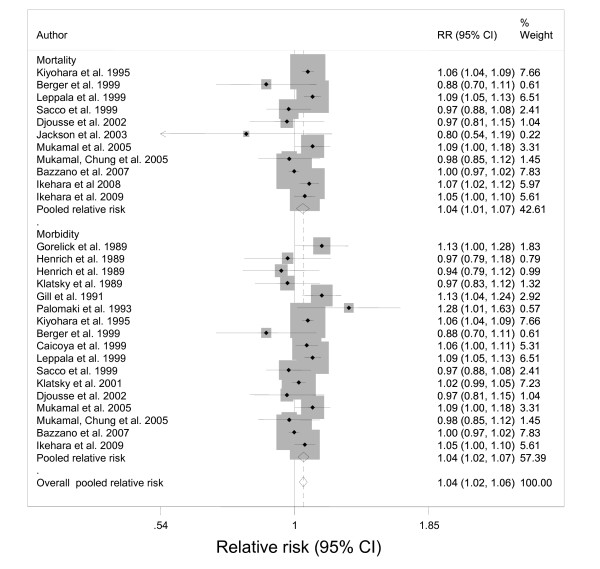
**Forest plot of risk estimates for alcohol consumption related to ischemic stroke of men by endpoint (18 studies)**.

**Figure 5:**
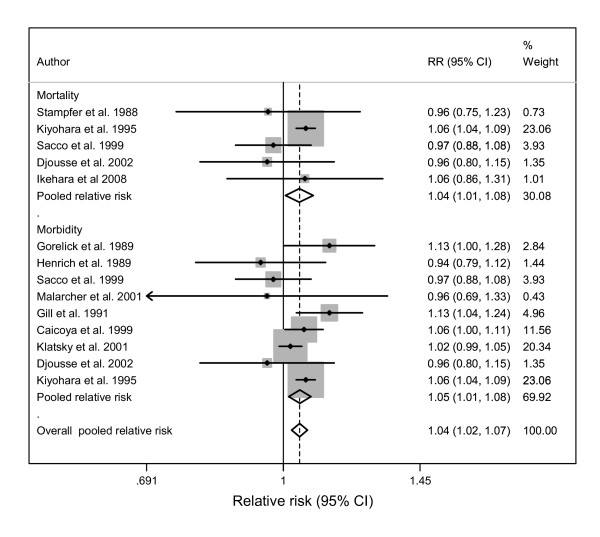
**Forest plot of risk estimates for alcohol consumption related to ischemic stroke of women by endpoint (11 studies)**.

In terms of morbidity, there were significant heterogeneities found among both men (Q-statistic(df) = 84.3(55), p = 0.0067; I^2 ^= 35, 95% CI: 9-53) and women (Q-statistic(df) = 45.5(26), p = 0.0104; I^2 ^= 43, 95% CI: 10-64) (See Figure 4). However, there was no publication bias detected by any methodology. For ischemic stroke mortality, there were no significant heterogeneities found among men (Q-statistic(df) = 40.0(36), p = 0.2966; I^2 ^= 10, 95% CI: 0-40) or women (Q-statistic(df) = 15.5(14), p = 0.3436; I^2 ^= 10, 95% CI: 0-47) (See Figures 5). Again, there was no publication bias.

### Dose-response relationship

**Figure 6:**
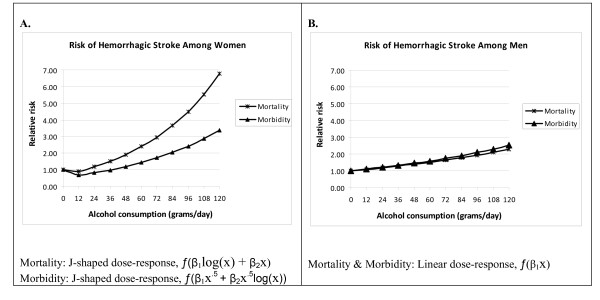
**Meta-analysis showing the dose-response relationship between alcohol and hemorrhagic stroke by sex and by endpoint**.

**Figure 7:**
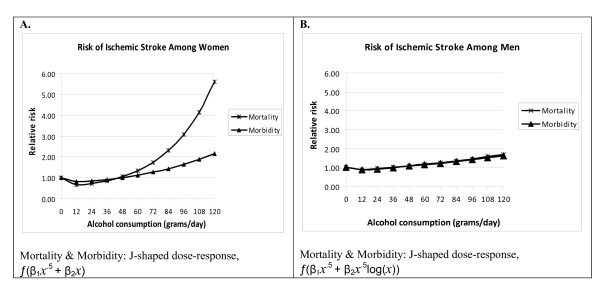
**Meta-analysis showing the dose-response relationship between alcohol and ischemic stroke by sex and by endpoint**.

A total of 44 models (8 first-order models and 36 second-order fractional polynomials), were examined for each stroke (ischemic and hemorrhagic) by sex and outcome. Figures 6 and 7 show the fitted curves of the dose-response relationship between alcohol and stroke subtypes by sex and by end point.

#### Haemorrhagic stroke

The overall results indicated a positive association between heavy alcohol consumption and RR of hemorrhagic-stroke mortality, irrespective of sex (Figure 6). Compared to the reference group of lifetime abstainers, at 96 grams (8 US standard drinks) of pure alcohol per day, RRs of 1.94 (95% CI: 1.56-2.40) and 4.50 (95% CI: 2.47-8.20) were calculated for men and women, respectively. Similarly, the risk of hemorrhagic-stroke morbidity for men also resulted in a monotonic relationship, almost linear on a logarithmized scale (RR = 2.52 at 10 drinks per day, 95% CI: 1.74-3.64). For women, the curve was J-shaped; there was a protective effect of moderate drinking up to 36 grams of pure alcohol or about 3 drinks a day. The nadir was reached at less than 1 drink per day (RR: 0.69, 95% CI: 0.54-0.89) (also see Additional file 3).

#### Ischemic stroke

On the other hand, an overall nonlinear association between alcohol consumption and RR of ischemic-stroke was observed. The association between alcohol consumption and RR of ischemic-stroke mortality had a J-shaped relationship irrespective of sex or end points (Figure 7). Relative to the lifetime male abstainers, alcohol consumption of less than 35 grams/day, or less than 3 drinks per day based on US conversions, was significantly associated with a decreased RR of ischemic-stroke. The risk curve had a global minimum (RR = 0.86, 95% CI: 0.81-0.93) for 12 grams or pure alcohol or 1 drink per day. For women, the lowest risk of mortality was among those consuming less than 12 grams/day, or about 1 drink/day. Protective effects were evident up to 44 grams/day (almost 4 drinks per day) (RR of 5.61, 95% CI: 3.12-10.09 for 12 standard drinks a day) (also see Additional file 3).

Similarly, the risk of ischemic-stroke morbidity for both sexes resulted in a J-shaped curve as well. There was a protective effect up to 37 grams/day (about 3 drinks/day) among men and 46 grams/day (or about 4 drinks/day) among women observed. For 12 drinks/day, the risk of ischemic-stroke morbidity was highest at RR = 1.60 (95% CI: 1.38-1.86) among men and RR = 2.15 (95% CI: 1.62-2.86) among women.

To assess whether the heterogeneity of study results may have been due to differences in the study's endpoint of stroke mortality or morbidity, we created an interaction (product) term between the type of endpoint under consideration (1 for mortality and 0 for morbidity) and the dose variable (alcohol consumption), and included it in the model for each sex. The interaction term for women was significant in both hemorrhagic (X^2 ^= 6.06, df = 1, p = 0.0138) and ischemic stroke (X^2 ^= 8.52, df = 1, p = 0.0141), suggesting that the effect of alcohol consumption was different in mortality compared with morbidity studies (See Figures 6a and 7a). However, the interaction term was not significant among men.

The findings from the sensitivity analyses based on different exclusion criteria are presented in Additional file 4. Risk estimates changed very little after the exclusion of: a) studies without computed tomography (CT) scans or other imaging measures; b) studies that did not adjust for important confounders (age, smoking and hypertension); c) or studies fulfilling criteria a) or b).

## Discussion

Overall, the present findings suggest a consistent association between heavy drinking and risk for stroke subtypes, whereas the evidence linking moderate consumption (1-2 drinks/day) appears to have mixed results.

The curvilinear relationship for ischemic stroke resembles the dose-response relationship between alcohol and ischemic heart disease [52] with the same postulated underlying biological mechanisms [53-55]. For hemorrhagic stroke, hypertension plays a more prominent role in the etiology. Alcohol, already operant at low doses, has been identified as a major risk factor for hypertension [56], and might explain the different dose-response relationship [45,48].

One of the main results of this study is the difference between mortality and morbidity as an endpoint, especially for women. While higher effects of alcohol on mortality compared to morbidity have been demonstrated for other chronic disease (e.g., liver cirrhosis [57] and injury [50], most meta-analyses in this area do not differentiate between these endpoints. Both for questions of etiology and prevention, however, such a distinction is necessary.

We conducted a meta-regression as a sensitivity analysis for each stroke type to measure the impact of study type (case control vs. cohort) as a potential covariate on the shape of the curve. We found that the shape of association remained unchanged for both hemorrhagic (p = 0.170) and ischemic stroke (p = 0.589) (data on RR not shown).

This analysis is subject to general strengths and limitations of meta-analyses, in addition to some subject-specific issues. Of the former, a major limitation is that the quality of our study depends on data from original publications included in our analysis. Our study may thus inherit some problems of potential bias and confounding effects associated with observational studies. However, a randomized controlled trial of alcohol consumption and stroke has not been performed and is highly unlikely to be conducted in the future for ethical reasons. Consequently, we must rely on data from observational studies to draw conclusions and make recommendations.

Of the latter, a limitation is that CT scans and other imaging techniques were not available for some of the studies [5,14,19,22,35,36,38,58]. At present, a CT scan is the most reliable method of distinguishing between hemorrhagic and ischemic strokes [59,60], but unfortunately these studies primarily used autopsy report, death certificates, or death registry data to make diagnoses and determine the outcome of stroke subtypes. However, given that the dose-response curve differed by type of stroke, any bias introduced would dilute such a difference. Additionally, our sensitivity analysis showed little difference as the shape of association remained unchanged.

Second, the selection of the reference group may vary among studies [61]. For instance, some studies used the lowest consumption level as the reference group while others used abstainers. It has been suggested that the non-linear association of between alcohol consumption and mortality from cardiovascular diseases could be due to the inclusion of ex-drinkers in the referent group of abstainers [25,62,63]. To avoid combining studies that were not comparable, we chose to correct the RR from studies based on current abstention by introducing the effect for former drinkers based on a meta-analyses of the studies where former drinkers were separated from lifetime abstainers. We calculated this correction for fatal and non-fatal outcomes combined to achieve more stable results, which may have biased the current results. However, our estimates of about 21% of males and 4% of females being ex-drinkers falls in line with the literature, and may even be an underestimate, meaning the results of this study would be conservative.

Finally, assessment methods for alcohol consumption may vary among studies. Alcohol consumption is usually measured by self-reported alcohol drinking habits. Such data are subject to recall bias. For example, heavy drinkers may be more likely to underreport their alcohol consumption or respondents simply forget about their consumption in retrospective recalls, which tends to result in an underestimation of alcohol consumption [64-66].

## Conclusions

Overall, our study showed differential impact of alcohol consumption on both type and outcome of stroke. Clearly, to reduce the risk of stroke, any heavy consumption of alcohol should be avoided. With respect to moderate consumption of up to 3 drinks, the results are mixed: moderate consumption seem to be protective for ischemic stroke only, but slightly detrimental or at best neutral for hemorrhagic stroke. In line with the results on the cardio-protective effect of alcohol including the evidence on biological pathways [53,67], it seems reasonable that the protective effect on ischemic stroke is limited to people who not only on average drink moderately, but who also avoid heavy drinking occasions [55].

Finally, we would like to point out the implications of our findings for global public health. With stroke currently being one of the most important causes of death and burden of disease (see above), and with the exposure to alcohol projected to increase proportionately to/with increases in wealth [68], alcohol-attributable stroke burden will continue to increase globally without an effort to increase effective alcohol control measures [69]. Overall, we recognize that total abstention, while beneficial for some stroke subtype, is advocated to reduce total mortality and morbidity, including that from injury. For the cardio-protective effects, however, we recommend that drinking alcohol be limited to 2 or less drinks per day.

## Competing interests

The authors declare that they have no competing interests.

## Authors' contributions

JP participated in the design of the study and carried out the literature review, statistical analysis, and drafted the manuscript. BT MR, DB and SM participated in the design of the study and helped in drafting the manuscript. HI helped in the statistical analysis. JR conceived of the study, and participated in its design of the study and helped to draft the manuscript. All authors read and approved the final manuscript.

## Pre-publication history

The pre-publication history for this paper can be accessed here:

http://www.biomedcentral.com/1471-2458/10/258/prepub

## Supplementary Material

Additional file 1 **Characteristics of 9 Case-Control Studies of Alcohol Consumption and Risk of Stroke subtypes**. Contains a table showing characteristics of Case-Control Studies of Alcohol Consumption and Risk of Stroke subtypes Click here for file

Additional file 2 **Characteristics of 17 Cohort Studies of Alcohol Consumption and Risk of Stroke subtypes**. Contains a table showing characteristics of Cohort Studies of Alcohol Consumption and Risk of Stroke subtypes Click here for file

Additional file 3 **Relative Risk (95% Confidence Interval) of Stroke Types Associated With Alcohol Consumption (1 standard US drink = 12 grams) by sex and endpoint (lifetime abstention was used as referent)**. Contains a table showing relative risks of stroke types associated With Alcohol Consumption by sex and endpoint Click here for file

Additional file 4 **Relative Risk (95% Confidence Interval) of Stroke Types Associated With Alcohol Consumption (1 standard US drink) According to Different Exclusion Criteria (lifetime abstention was used as referent)**. Contains a table showing relative risks of stroke types associated With Alcohol Consumption according to exclusion criteria Click here for file
