# Supplementary material for: Alcohol consumption and the risk of morbidity and mortality for different stroke types - a systematic review and meta-analysis
Source: BMC Public Health. 2010 May 18;10:258. doi: 10.1186/1471-2458-10-258 (PMC2888740; doi:10.1186/1471-2458-10-258)
Supplement: Additional file 1 — Characteristics of 9 Case-Control Studies of Alcohol Consumption and Risk of Stroke subtypes. Contains a table showing characteristics of Case-Control Studies of Alcohol Consumption and Risk of Stroke subtypes [file 1471-2458-10-258-S1.DOC]

**Table 1.** Characteristics of 9 Case-Control Studies of Alcohol Consumption and Risk of Stroke subtypes

| **Source** | **Stroke cases** | **Controls** | **Case assessment** | **Exposure assessment** | **Age at baseline** | **Covariate List** | **Stroke Subtype** | **End Point** |
| --- | --- | --- | --- | --- | --- | --- | --- | --- |
| Gorelick etal 1989 | 201 MW patients with incident ischemic stroke in 3 medical centers in Chicago | 405 Outpatient clinic patients | Clinical diagnosis and CT scan | Self-administered | n/a | Age, sex, smoking, hypertension | Ischemic stroke | Morbidity |
| Henrich etal 1989 | 82 MW patients; USA | 174 patients | Clinical diagnosis and head CT | Interviewer administered | 57 | Age, hypertension, diabetes, smoking | Ischemic stroke | Morbidity |
| Gill etal 1991 | 624 MW hospitalized patients with stroke diagnosis in 2 centres in UK | 573 MW industrial workers in the same community | Clinical examination, CT scan, angiography and postmortem examination, or lumbar puncture | Interviewer administered | 49 | Age sex, race,SES, TxH, smoking, medication | Ischemic and Hemorrhagic stroke | Morbidity |
| Longstreth etal 1992 | 149 MW patients from King County, Washington | 298 controls randomly selected | CT scan | Structured in-person interview | 55 | Age, gender | Hemorrhagic stroke | Mortality & Morbidity |
| Palomaki etal 1993 | 156 M hospitalized patients with ischemic stroke in Finland | 153 Hospital patients | Clinical diagnosis | Interviewer administered | 49 | Age, arterial hypertension, CHD, smoking, bmi, diabetes, history of snoring | Ischemic stroke | Morbidity |
| Caicoya etal 1999 | 467 MW patients with incident stroke in Spain | 477 Residents of the same community | Clinical examination or CT scan | Interviewer administered | 63 | Age, smoking, hypertension, hypercholesterolemia, diabetes, cardiac disease | Ischemic and Hemorrhagic stroke | Morbidity |
| Sacco etal 1999 | 677 MW with incident cerebral infarction in the community in New York | 1139 Community controls | Brain imaging and clinical diagnosis | Interviewer administered | 70 | Hypertension, diabetes, cardiac diasease, smoking, education, bmi | Ischemic stroke | Mortality & Morbidity |
| Thrift etal 1999 | 294 MW patients with primary hemorrhagic stroke from 13 hospitals in Melbourne, Australia | 306 Residents from the same neighborhood | CT scan, MRI, or autopsy | Interviewer administered | 63 | Cholesterol, previous CVD, diabetes, exercise, bmi, smoking, education | Hemorrhagic stroke | Mortality & Morbidity |
| Malarcher etal 2001 | 191 W patients with incident cerebral infarction in 59 hospitals in Baltimore-Washington region in the United States | 379 W community residents | Hospital discharge diagnosis, clinical diagnosis, neuro-imaging results, or autopsy reports | Interviewer administered | 35 | Age, race, education, smoking, bmi, total cholesterol, HDP cholesterol, hypertension, CHD, diabetes | Ischemic stroke | Morbidity |

*Abbreviations: M Men; W Women; TxH Treatment for hypertension; BMI, Body mass index; CT, computer tomography; DM, diabetes mellitus; MI, myocardial infarction; MRI, magnetic resonance imaging; SES, socioeconomic status; TIA, transient ischemic attack.*
