# Supplementary material for: Alcohol consumption and the risk of morbidity and mortality for different stroke types - a systematic review and meta-analysis
Source: BMC Public Health. 2010 May 18;10:258. doi: 10.1186/1471-2458-10-258 (PMC2888740; doi:10.1186/1471-2458-10-258)
Supplement: Additional file 2 — Characteristics of 17 Cohort Studies of Alcohol Consumption and Risk of Stroke subtypes. Contains a table showing characteristics of Cohort Studies of Alcohol Consumption and Risk of Stroke subtypes [file 1471-2458-10-258-S2.DOC]

**Table 2.** Characteristics of 17 Cohort Studies of Alcohol Consumption and Risk of Stroke subtypes

| **Source** | **Country** | **Study participants** | **Exposure assessment** | **Duration of follow up in yrs** | **Follow up process** | **Outcome assessment** | **No. of cases** | **Covariate**  **List** | **Stroke**  **Type** | **End Point** |
| --- | --- | --- | --- | --- | --- | --- | --- | --- | --- | --- |
| Donahue etal 1986 | USA | 7878 M aged 45-69 y | Interviewer administered | 12 | Clinical examinations at years 2 and 6 and continued surveillance | Hospital discharge diagnosis, clinical diagnosis, death certificate, or autopsy record | 290 | Age, BMI, cigarette smoking, hypertension, serum cholesterol, uric acid, glucose level, hematocrit | Hemorrhagic stroke | Mortality and morbidity |
| Kono etal 1986 | JAPAN | 5135 M in Japan | Self-administered questionnaire | 19 | Vital status ascertained by medical association | Death certificate | 230 | Age, cigarette smoking | Hemorrhagic stroke | Mortality |
| Stampfer etal 1988 | USA | 87 526 US W aged 34-59 y | Self-administered questionnaire | 4 | Biennial questionnaires | Medical records | 120 | Age, cigarette smoking, hypertension, DM, serum cholesterol level, obesity, exercise, cholesterol intake, saturated and polyunsaturated fat intake, parental istory of MI before age 60 y, menopausal status, hormone use, study period | Ischemic and Hemorrhagic stroke | Mortality |
| Klatsky etal 1989 | USA | 107 137 US MW aged 50 y | Self-administered questionnaire | 6 | Surveillance of hospital discharges | Clinical diagnosis | 674 | Age, sex, race, cigarette smoking, SBP, coffee consumption, BMI, baseline disease | Ischemic and Hemorrhagic stroke | Morbidity |
| Kiyohara etal 1995 | Japan | 1621 MW aged 40 y in Japan | Interviewer administered | 26 | Biennial examinations, mail, or telephone | Neurological examination, CT scan, angiography, lumbar puncture, or autopsy | 304 | Age, sex, hypertension | Ischemic and Hemorrhagic stroke | Both mortality and morbidity |
| Berger etal 1999 | USA | 21870 M physicians aged 40-84 y in Physicians`Health Study | Mailed questionnaire | 12.2 | Questionnaires collected bi-annually at first year then annually thereafter;  alcohol consumption reassessed at 84 months of followup | Medical records, death certificate, brain imaging, judgment of neurologist | 679 | SBP, treatment for hypertension, smoking, history of diabetes, BMI, exercise | Ischemic and Hemorrhagic stroke | Both mortality and morbidity |
| Leppälä etal 1999 | Finland | 26 556 M aged 50-69 y in Finland | Self-administered questionnaire | 6.1 | National hospital discharge register and national death register | Clinical diagnosis or death certificate | 960 | Age, BMI, cigarette smoking, serum cholesterol level, DM, educational level, leisure time physical activity, heart disease, supplementation with  -tocopherol or beta carotene | Ischemic and Hemorrhagic stroke | Both mortality and morbidity |
| Sankai etal 2000 | Japan | 12372 MW aged 40-69 y in 6 Japanese communities (5 rural, 1 suburban) | Interviewer administered | 9.4 | Cardiovascular disease surveillance | CT and/or clinical diagnosis; death certificates | 23 | Age, sex, smoking, BP, serum total cholesterol, BMI, diabetes | Hemorrhagic stroke | Both mortality and morbidity |
| Klatsky etal 2001 | USA | 128,934 members of prepaid health care program in San Francisco and Oakland | Health examination questionnaire | 7 | Followed up until end of 1996 or death or health plan termination or first hospitalization | Chart review, death certificate | 1566 | Age, sex, race, BMI, education, smoking | Ischemic stroke | Morbidity |
| Suh etal 2001 | Korea | 114,793 M aged 35-59 y in 1990-92 | Self-administered questionnaire | 6 | For individuals who had more than one  stroke follow up occurred until first event; individuals were followed up for 6 years | Chart review, death certificate | 372 | No covariates | Hemorrhagic stroke | Mortality |
| Djousse etal 2002 | USA | 5209 MW Framingham, Mass. | Interviewer administered | 30 | Biennial examinations | Clinical diagnosis and radiographic images | 441 | Age, BMI, cigarette smoking, DM | Ischemic stroke | Both mortality and morbidity |
| Jackson etal 2003 | USA | 1320 M physicians with history of stroke aged 40-84 years identified from mailing lists of AMA, Chicago | Self-reported data on alcohol consumption | 4.5 | Questionnaires collected biennially at first year then annually thereafter | National death index | 145 | Age, smoking, diabetes, BMI, exercise, angina, MI | Ischemic stroke | Mortality |
| Mukamal etal 2005 | USA | 38156 M health professionals | Follow-up questionnaires were sent biennially | 14 | Follow-up questionnaires were sent biennially | Reported by families, postal officials and through National Death Index | 412 | Age, smoking, BMI, geographic region, parental history of MI, physical activity, hypercholesterolemia, aspirin use, diabetes, intake of Vit E, folate, energy, saturated fat, trans fat, potassium, magnesium, w-3 fatty acids and dietary fibre, hypertension | Ischemic stroke | Mortality and morbidity |
| Mukamal, Chung etal 2005 | USA | 4410 MW Cardiovascular health study aged above/or 65 y in 4 US communities | Standardized questionnaire | 9.2 | At baseline and annually until 1999 | Imaging studies, medical records | 434 | Age, sex, race, education, marital status, smoking, exercise, depression, aspirin use, BMI, diabetes at baseline, SBP | Ischemic stroke | Both mortality and morbidity |
| Bazzano etal 2007 | China | 64,338 M aged >=40 y free of stroke 1991 | Interviewer administered | 9 | Follow-up in 1999-2000 | Tomography, MRI, autopsy, laboratory results, discharge diagnoses | 3434 | Age, BMI, physical activity, geographic region, smoking, diabetes, education, SBP | Ischemic and Hemorrhagic stroke | Mortality and morbidity |
| Ikehara etal 2008 | Japan | 34,776 M and 48,906 W aged 40 to 79 years | Self-administered questionnaire | 14.2 | Followed up between 1993 and 2003 | Systematic review of death certificate | 864 | Age, smoking, bmi, hypertension, history of diabetes, exercise, mental stress, education, vegetable intake, fish, fruit | Ischemic and Hemorrhagic stroke | Mortality |
| Ikehara etal 2009 | Japan | Prospective data for 19,356 M aged 40-69 y in 1993 | Self-administered questionnaire | 10 | Followed up between 1993 and 2003 | CT, MRI, autopsy | 629 | Age, smoking, BMI, diabetes, education, sports at leisure time, fruits, vegetable, fish intake, hypertension, and public health sites | Ischemic and Hemorrhagic stroke | Both mortality and morbidity |

*Abbreviations: M Men; W Women; BMI, body mass index; CT, computed tomography; DBP, diastolic blood pressure; DM, diabetes mellitus; ECG, electrocardiogram; FEV, forced expiratory volume; MI, myocardial infarction; NHS, National Health Service; SBP, systolic blood pressure.*
