# Supplementary material for: Alcohol consumption and the risk of morbidity and mortality for different stroke types - a systematic review and meta-analysis
Source: BMC Public Health. 2010 May 18;10:258. doi: 10.1186/1471-2458-10-258 (PMC2888740; doi:10.1186/1471-2458-10-258)
Supplement: Additional file 3 — Relative Risk (95% Confidence Interval) of Stroke Types Associated With Alcohol Consumption (1 standard US drink = 12 grams) by sex and endpoint (lifetime abstention was used as referent). Contains a table showing relative risks of stroke types associated With Alcohol Consumption by sex and endpoint [file 1471-2458-10-258-S3.DOC]

**Table 3.** Relative Risk (95% Confidence Interval) of Stroke Types Associated With Alcohol Consumption (1 standard US drink=12 grams) by sex and endpoint (lifetime abstention was used as referent)

|  |  | **Alcohol intake, No. of drinks/day** | | | | | | | |
| --- | --- | --- | --- | --- | --- | --- | --- | --- | --- |
|  |  | **1 drink** | | **3 drinks** | | **5 drinks** | | **7 drinks** | |
| **Stroke Type** | **Number of studies** | **Mortality** | **Morbidity** | **Mortality** | **Morbidity** | **Mortality** | **Morbidity** | **Mortality** | **Morbidity** |
| **Ischemic†** | **20** | 0.86  (0.80-0.92) | 0.86  (0.81-0.92) | 1.00  (0.93-1.07) | 0.99  (0.92-1.06) | 1.17  (1.08-1.26) | 1.14  (1.05-1.24) | 1.36  (1.23-1.51) | 1.31  (1.18-1.46) |
| M | 18 | 0.86  (0.81-0.93) | 0.87  (0.81-0.93) | 1.00  (0.94-1.07) | 0.99  (0.92-1.07) | 1.17  (1.09-1.27) | 1.15  (1.05-1.25) | 1.36  (1.23-1.50) | 1.32  (1.18-1.47) |
| F | 11 | 0.66  (0.55-0.79) | 0.82  (0.74-0.92) | 0.85  (0.73-1.00) | 0.92  (0.81-1.05) | 1.35  (1.14-1.60) | 1.13  (0.98-1.31) | 2.31  (1.70-3.13) | 1.44  (1.19-1.74) |
| **Hemorrhagic‡** | **16** | 1.01  (1.01-1.02) | 0.90  (0.78-1.05) | 1.12  (1.08-1.16) | 1.15  (0.97-1.38) | 1.37  (1.25-1.51) | 1.45  (1.18-1.80) | 1.87  (1.55-2.25) | 1.81  (1.40-2.33) |
| M | 15 | 1.09  (1.06-1.12) | 1.10  (1.06-1.14) | 1.28  (1.18-1.39) | 1.32  (1.18-1.47) | 1.51  (1.32-1.73) | 1.59  (1.32-1.91) | 1.79  (1.48-2.15) | 1.91  (1.47-2.47) |
| F | 8 | 0.89  (0.52-1.52) | 0.69  (0.54-0.89) | 1.52  (1.08-2.14) | 0.99  (0.73-1.33) | 2.39  (1.61-3.55) | 1.43  (0.99-2.05) | 3.66  (2.16-6.19) | 2.03  (1.30-3.18) |

**†^[4],[8],[9],[10], [14], [16], [18], [19], [20], [21], [23], [24], [33],[34], [35], [36], [37], [39], [40], [41]^**

**‡ ^[4], [5], [6], [9], [15], [16], [17], [19], [22], [23], [24], [33], [34], [36], [38], [39]^**
