# Supplementary material for: Alcohol consumption and the risk of morbidity and mortality for different stroke types - a systematic review and meta-analysis
Source: BMC Public Health. 2010 May 18;10:258. doi: 10.1186/1471-2458-10-258 (PMC2888740; doi:10.1186/1471-2458-10-258)
Supplement: Additional file 4 — Relative Risk (95% Confidence Interval) of Stroke Types Associated With Alcohol Consumption (1 standard US drink) According to Different Exclusion Criteria (lifetime abstention was used as referent). Contains a table showing relative risks of stroke types associated With Alcohol Consumption according to exclusion criteria [file 1471-2458-10-258-S4.DOC]

**Table 4.** Relative Risk (95% Confidence Interval) of Stroke Types Associated With Alcohol Consumption (1 standard US drink) According to Different Exclusion Criteria (lifetime abstention was used as referent)

|  |  | **Alcohol intake, No. of drinks/day** | | | | | | | |
| --- | --- | --- | --- | --- | --- | --- | --- | --- | --- |
|  |  | **1 drink** | | **3 drinks** | | **5 drinks** | | **7 drinks** | |
| **Studies included in analyses by stroke type** | **# of studies** | **Mortality** | **Morbidity** | **Mortality** | **Morbidity** | **Mortality** | **Morbidity** | **Mortality** | **Morbidity** |
| **Ischemic** | 20 | 0.86  (0.80-0.92) | 0.86  (0.81-0.92) | 1.00  (0.93-1.07) | 0.99  (0.92-1.06) | 1.17  (1.08-1.26) | 1.14  (1.05-1.24) | 1.36  (1.23-1.51) | 1.31  (1.18-1.46) |
| a) Studies that used computed tomography scans or other imaging measures as an outcome measure† | 17 | 0.87  (0.80-0.93) | 0.87  (0.81-0.93) | 1.00  (0.93-1.08) | 1.01  (0.93-1.09) | 1.16  (1.06-1.26) | 1.17  (1.07-1.29) | 1.33  (1.19-1.49) | 1.36  (1.20-1.54) |
| b) Studies that controlled for important stroke risk factors (age, smoking, hypertension)‡ | 14 | 0.84  (0.77-0.91) | 0.86  (0.79-0.94) | 0.94  (0.87-1.02) | 0.98  (0.88-1.09) | 1.08  (0.97-1.19) | 1.13  (0.99-1.30) | 1.22  (1.07-1.40) | 1.30  (1.09-1.54) |
| Studies that had both a & b**^§^** | 12 | 0.85  (0.78-0.93) | 0.86  (0.79-0.94) | 0.93  (0.85-1.02) | 0.98  (0.88-1.09) | 1.04  (0.93-1.16) | 1.13  (0.99-1.30) | 1.15  (0.99-1.34) | 1.30  (1.09-1.54) |
| **Hemorrhagic** | 16 | 1.01  (1.01-1.02) | 0.90  (0.78-1.05) | 1.12  (1.08-1.16) | 1.15  (0.97-1.38) | 1.37  (1.25-1.51) | 1.45  (1.18-1.80) | 1.87  (1.55-2.25) | 1.81  (1.40-2.33) |
| a) Studies that used computed tomography scans or other imaging measures as an outcome measure†† | 13 | 1.01  (1.01-1.02) | 0.90  (0.78-1.05) | 1.12  (1.07-1.18) | 1.15  (0.97-1.38) | 1.37  (1.20-1.57) | 1.45  (1.18-1.80) | 1.86  (1.43-2.41) | 1.81  (1.40-2.33) |
| b) Studies that controlled for important stroke risk factors (age, smoking, hypertension)‡‡ | 8 | 1.01  (1.01-1.01) | 0.88  (0.75-1.04) | 1.10  (1.06-1.14) | 1.04  (0.86-1.26) | 1.30  (1.18-1.43) | 1.23  (0.98-1.54) | 1.67  (1.38-2.03) | 1.44  (1.09-1.89) |
| Studies that had both a & b**^§§^** | 7 | 1.01  (1.00-1.01) | 0.88  (0.75-1.04) | 1.09  (1.04-1.14) | 1.04  (0.86-1.26) | 1.27  (1.12-1.43) | 1.23  (0.98-1.54) | 1.59  (1.25-2.02) | 1.44  (1.09-1.89) |

**† ^[4], [8], [9], [10], [16], [18], [19], [20], [21], [24], [33], [34], [36], [37], [38], [40], [41]^**

**‡ ^[4], [8], [9], [10], [18], [20], [23], [24], [33], [34], [35], [37], [40], [41]^**

**§ ^[4], [8], [9], [10], [18], [20], [24], [33], [34], [37], [40], [41]^**

**†† ^[4], [5], [6], [9], [15], [16], [17], [19], [24], [33], [34], [36], [38]^**

**‡‡  ^[4], [5], [9],[15], [23], [24], [33], [34]^**

**§§ ^[4],[5],[9],[15],[24] [33],[34]^**
